# Nanoindentation Study on the Creep Characteristics and Hardness of Ion-Irradiated Alloys

**DOI:** 10.3390/ma13143132

**Published:** 2020-07-14

**Authors:** Zhenbo Zhu, Hefei Huang, Jizhao Liu, Linfeng Ye, Zhiyong Zhu

**Affiliations:** 1 Shanghai Institute of Applied Physics, Chinese Academy of Sciences, Shanghai 201800, China; zhuzhenbo@sinap.ac.cn (Z.Z.); liujizhao@sinap.ac.cn (J.L.); yelinfeng@sinap.ac.cn (L.Y.); 2 School of Nuclear Science and Technology, University of Chinese Academy of Sciences, Beijing 100049, China

**Keywords:** Xe ion irradiation, nanoindentation, irradiation induced hardening, creep plasticity

## Abstract

The Hastelloy N alloy, Alloy 800H and 316H stainless steel were irradiated by Xe^20+^ ion irradiation with energy of 4 MeV at room temperature (peak damage ranging from 0.5 to 10 dpa). The micromechanical properties, hardness and creep plasticity, of these three investigated alloys were characterized before and after irradiation using nanoindentation. The results show that the hardness increases, and creep plasticity degrades with increasing ion dose in all the samples. In comparison, Hastelloy N has good irradiation damage resistance, while that of the 800H and 316H alloys is slightly worse. Additionally, the approximate positive relationship between irradiation hardening and creep plasticity degradation means that the property of creep plasticity of irradiated materials can be reflected from the nanohardness measurement for the heavy ion irradiation cases.

## 1. Introduction

Molten salt reactors (MSRs) are one of the six most advanced fission reactors for the future generation of electricity and heat to achieve hydrogen production [1,2]. The nickel based Hastelloy N alloy is considered to be the most promising candidate structural material for MSRs operated at 650 °C because of its excellent resistance to molten fluoride salt [3]. In order to make the most of MSRs’ advantages such as effective hydrogen production, the operation temperature needs to exceed 700 °C [4]. Considering that the maximum allowable stress of Hastelloy N alloy over 700 °C may become too low to poses a threat to the safe operation of reactors [1], screening appropriate structural materials combining high-temperature strength, chemical compatibility with the liquid fluoride salt and resistance to high-flux neutron irradiation is a key issue related to the development of a higher-temperature MSRs. Since the irradiation damage [5,6] caused by neutron bombardment in the reactor environment can greatly degrade the mechanical properties of structural materials, the evaluation of variation in mechanical properties caused by the irradiation damage is one of great importance for the safe operation of MSRs.

The Alloy 800H and 316H stainless steel, are regarded as the candidate structural materials for the design and construction of generation IV fission reactors as well [7,8]. Currently, the Alloy 800H is considerate for use in nuclear systems with operation temperature up to 760 °C owing to its high temperature strength and good resistance to swelling, corrosion and creep rupture [7,9]. As for the 316H stainless steel, the version with high carbon content, features the advantages of low cost and high temperature mechanical properties, with a maximum temperature of design stress intensity up to 825 °C [10]. In order to further evaluate their application prospects in the high-temperature MSRs, it is necessary to study the irradiation resistance of these materials.

Due to the limited access of neutron irradiation facilities, ion beam irradiation has become an attractive tool to investigate irradiation damage with negligible induced-radioactivity and high usability. Since its shallow implantation depth using accelerator-based ion beam, it is difficult to perform macroscopic characterizations of ion irradiated alloys. Numerous studies focusing on microstructural evolution of pure metals and alloys after irradiation have been performed using transmission electron microscope (TEM), X-ray diffraction (XRD), scanning electron microscope (SEM), etc. [11,12,13]. In recent years, with the application of nanoindentation technology, the hardening phenomenon of irradiated materials has been well characterized by testing the nanohardness value of the samples before and after irradiation [14,15,16]. Normally, this form of irradiation hardening is considered to be closely related to the irradiation embrittlement of materials, but the direct evidence of the relationship between hardening and embrittlement is rarely given. It worth noting that nanoindentation can also be used to probe the creep plasticity qualitatively, a characteristic parameter representing material embrittlement, of materials by sampling a very small volume. While holding the compression load during nanoindentation, materials experience continuous displacements, mimicking the conventional primary and secondary creep stages, from which similar creep parameters can are extractable [17]. Therefore, the establishment of the relationship between the hardness increase and the creep plasticity degradation of materials is expected to provide evidence for the relationship between irradiation hardening and embrittlement.

As one kind of fission products in MSRs, Xe ions can cause significant displacement damage within the material, which has similar damage behavior with neutron irradiation. The previous research results show that the irradiation hardening of nickel-based alloys occurred caused by the displacement damages both at room temperature (RT) and high temperature and it is more serious at former case [18]. As part of a series of studies, bulk samples of the Alloy 800H and 316H stainless steel were irradiated by Xe ions (up to 10 dpa) at room temperature (RT) in this study. In addition, the Hastelloy N alloy was selected as the reference sample. Both nanohardness and the creep plasticity of these investigated alloys were measured using nanoindentation to compare the plasticity and hardness of the investigated alloys after irradiation. Furthermore, the comparison of the hardness increment and creep plasticity degradation was also performed to investigate their relationship.

## 2. Materials and Experimental Procedure

### 2.1. Materials Preparation

The bulk samples used herein with size of 40 mm × 40 mm × 150 mm were the Hastelloy N alloy, Alloy 800H (denoted as 800H in table and graphs) and 316H stainless steel (denoted as 316H in table and graphs) and were subjected to cold-rolling and annealing treatments. All the alloys have the face-centered cubic (FCC) lattice structure and their chemical compositions are shown in Table 1. It is noted that the Hastelloy N alloy and Alloy 800H are nickel-based alloys, while the 316H stainless steel is iron-based alloy. Bulk samples of all three alloys with size of 5 mm × 5 mm × 1 mm were successively polished with SiC sandpaper and alumina suspension. After this, the polished samples were putted in a solution of H_2_SO_4_, glycerin and deionized water in a 5:4:1 ratio for 10 s at 36 V and 0 °C to remove surface stresses.

### 2.2. Ion Irradiation

**Figure 1:**
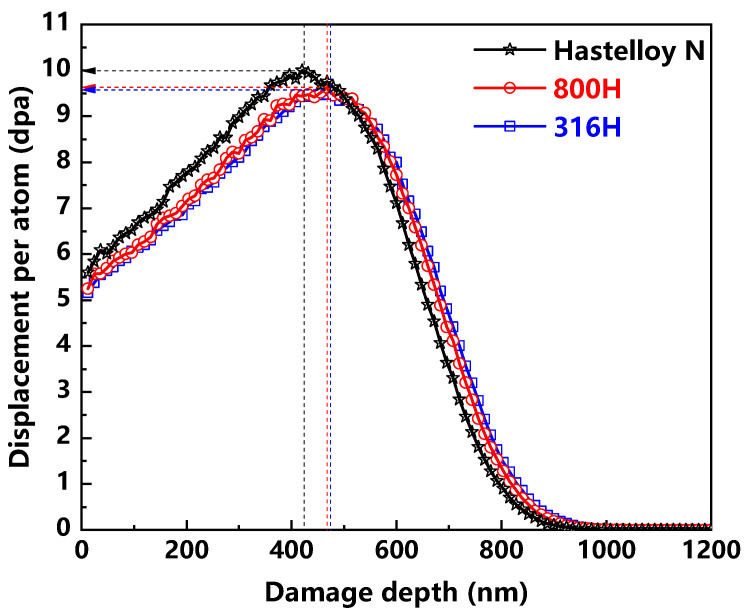
SRIM calculation of the damage profiles produced by 4MeV Xe^20+^ with the ion dose of 3.37 × 10^15^ ion/cm^2^ in the Hastelloy N alloy, Alloy 800H and 316H stainless steel.

The as-prepared samples of all the alloys were irradiated by Xe^20+^ ions at RT. The energy of Xe^20+^ ion was fixed at 4 MeV, and the ion doses used in this study were about 1.69 × 10^14^, 6.74 × 10^14^ and 3.37 × 10^15^ ions/cm^2^. The profiles of irradiation damages caused by Xe ion at the ion dose of 3.37 × 10^15^ ions/cm^2^ are shown in Figure 1. These profiles were calculated using the K–P quick calculation mode in SRIM-2013 software [19] assuming atomic displacement energy of 40 eV. The irradiation damages by 4 MeV Xe^20+^ ions for all the alloys are calculated to extend up to the depth about 1100 nm below the surface. In the case of ion dose of 3.37 × 10^15^ ions/cm^2^, the corresponding peak damages locate at around 420, 460 and 465 nm for the Hastelloy N alloy, Alloy 800H and 316H stainless steel, respectively. Moreover, their corresponding peak damages are 10, 9.6 and 9.6 dpa. It can be noted that the damage level caused by Xe ion with identical energy was approximately the same in the investigated samples, with the calculated error no more than 4%. As for the cases of ion dose of 1.69 × 10^14^ and 6.74 × 10^14^, the peak damages generated in three alloys are around 0.5 and 2 dpa, respectively.

### 2.3. Nanoindentation Characterization

After ion beam irradiation, nanoindentation was conducted on the all investigated samples surface using G200 nanoindenter to characterize their hardness and creep plasticity. The diamond Berkovich tip (model TB13989-XP) was adopted with a nominal radius of 20 nm under a continuous stiffness measurement (CSM) mode. The experimental hardness was determined by analyzing load–displacement (P–h) curves using the Oliver and Pharr method [20]. In order to ensure accuracy of the experimental results, 10 single indents were made for each sample. The critical indentation depths, whose influential zone covers all the irradiated plasticity affected region, can be obtained to serve as the constant load beginning depth in further indentation creep tests.

As for the creep tests, the indentation was performed using varying forces needed to achieve the desired depth using constant strain rate mode. In the following progress of holding constant load starting at a critical indentation depth, the indenter is continuously sinking into the material. Since the load is constant and the contact area is increasing during this process, the mean applied stress acting on the material is reducing as the creep continuously goes on. The relationship between applied stress and instantaneous indent tip depth is assessed to reveal the creep plasticity. To ensure the reliability of creep results, independent measurements in groups of 12 were conducted for each case at room temperature while strain rate was 0.1 s^−1^ and the holding time was 100 s. For each indent, a full load displacement curve as a function of time was recorded to obtain creep parameters (e.g., stress exponent).

## 3. Results and Discussion

### 3.1. Irradiation Induced Hardening

**Figure 2:**
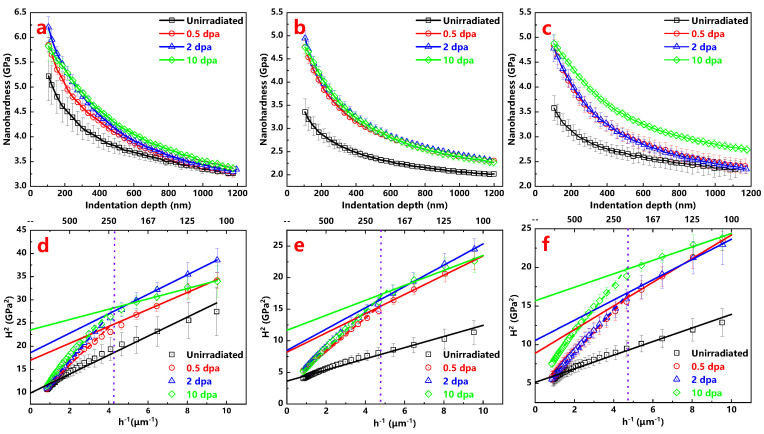
Profiles of average nanohardness in the function of the indentation depth of the (**a**) Hastelloy N alloy; (**b**) Alloy 800H; (**c**) 316H stainless steel before and after irradiation and (**d**–**f**) the corresponding curves of H^2^ versus 1/h.

Figure 2a–c show the average nanohardness of the Hastelloy N alloy, Alloy 800H and 316H stainless steel before and after ion irradiation as a function of the indentation depth. The data in the depths less than 100 nm were ignored due to uncertainty deriving from indenter tip complexity [21]. Compared with the unirradiated samples, the results of all the ion irradiated samples clearly show the presence of irradiation hardening. Using the Nix–Gao model can help evaluate the values of irradiation induced hardening and the model is described as following Equation [22]:

(1) $$H^{2}=H_{0}{}^{2}+\frac{H_{0}{}^{2}h^{*}}{h_{c}}$$

where H is the measured hardness at the depth of $h_{c}$, $H_{0}$ is the hardness at infinite depth, $h^{*}$ is the characteristic length which depends on the material and the shape of the indenter tip.

As shown in Figure 2d–f, the curves of $H^{2}$ versus $\frac{1}{h}$ of all the samples are plotted to obtain their values of hardness before and after irradiation based on the method reported by Kasada et al. [23]. Taking into account the error bar, the curves of the unirradiated samples have a good linearity above 100 nm, while those of the irradiated samples present the characteristic with approximate bilinearity. This mentioned above phenomenon is due to the softer substrate effect (SSE) [23]. In this study, the critical indentation depths are about 230, 210, 210 nm, which correspond to the Hastelloy N alloy, Alloy 800H, 316H stainless steel, respectively. Here, ratios of the radius of plasticity affected region to critical indentation depth are approximately 5, which is in agreement with previous study [24]. Moreover, these critical depths will serve as corresponding constant load beginning depth to obtain creep parameters in further nanoindentation creep tests.

**Figure 3:**
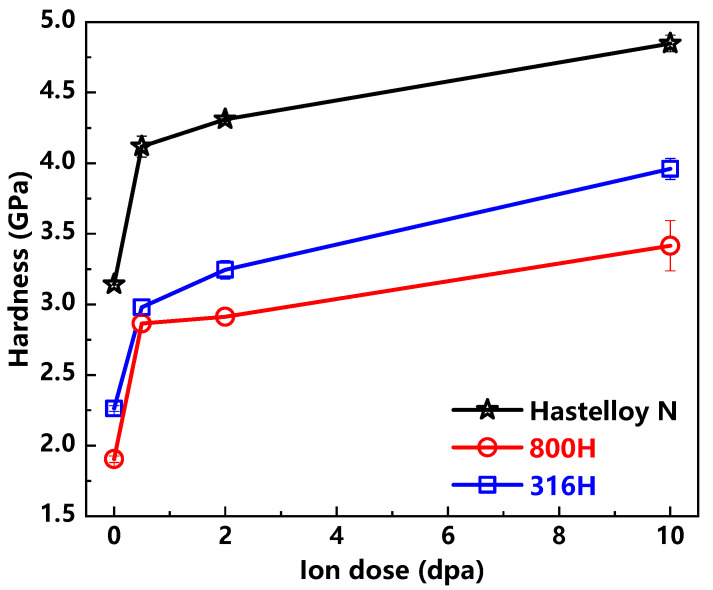
Experiment obtained H values versus ion dose of the Hastelloy N alloy, Alloy 800H and 316H stainless steel.

The nanohardness $H_{0}$ of the Hastelloy N alloy, Alloy 800H and 316H stainless steel calculated by Nix-Gao model are showed in Figure 3. The nanohardness values of the Alloy 800H and 316H stainless steel are similar, while this value of the Hastelloy N alloy is significantly higher than that of other two alloys by comparison. The changes of nanohardness $H_{0}$ show that the hardness of samples is increasing with the increase in ion dose. In addition, the hardness increments have the saturated tendencies for all the alloys. It is generally accepted that the ion irradiation induced defects, especially the dislocation loops, can act as obstacles for the free movement of the dislocation lines, thus resulting in the irradiation hardening [21,25]. In this study, although the characterization of the internal microstructural evolution of samples before and after irradiation has not been carried out, combined with previous research work [26,27,28], it can be reasonably inferred that irradiation damage defects are the cause of hardening of all three samples.

**Figure 4:**
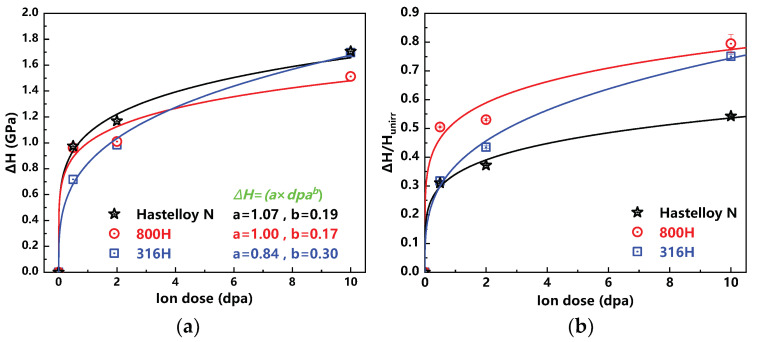
(**a**) Hardness increments (ΔH) versus ion dose of the Hastelloy N, Alloy 800H and 316H stainless steel; (**b**) ΔHHunirr versus ion dose of the Hastelloy N, Alloy 800H and 316H stainless steel.

Currently, an Equation, revealing the relationship between nanohardness and ion dose, is purposed and described as follows [29]:

(2) $$\Delta H=a\times dpa^{b}$$

where ΔH is the hardness increment of irradiated sample. *a* and *b* are fitting parameters. The profiles, as shown in Figure 4a, display the power-law dependence of hardness increment ΔH on ion dose in all the investigated alloys. The value of *b* for the Hastelloy N alloy is 0.19, which agrees well with previous study (~0.2) on the GH3535 alloy [18]. In addition, it can be found that the values *a* and *b* for the Alloy 800H are similar to that of the Hastelloy N alloy. That means the ΔH variation trend of the Alloy 800H is similar to that of the Hastelloy N alloy. As for the 316H, the value of *b* is 0.3, which means the nanohardness of the 316H increases faster than the other two alloys with the increase of ion dose. Considering the nanohardness difference of these three unirradiated alloys, ΔHHunirr is used to reveal the degree of irradiation induced hardening as shown in Figure 4b. It is not difficult to find that the hardening degree of the Alloy 800H is much higher than that of the Hastelloy N alloy, although their ΔH values are similar. The hardening degree of the 316H with the change of ion irradiation dose is between the other two alloys.

### 3.2. Nanoindentation Creep

**Figure 5:**
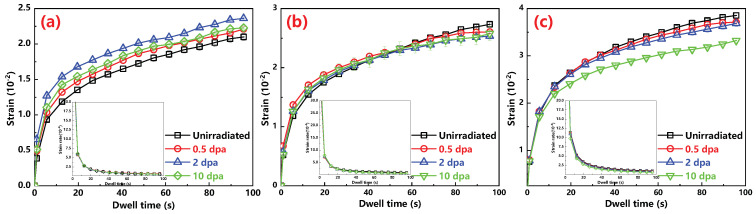
Strain and corresponding strain rate versus dwell time of the (**a**) Hastelloy N alloy; (**b**) Alloy 800H; (**c**) 316H stainless steel before and after ion irradiation.

The corresponding constant load beginning depth on the investigated alloys are obtained in the last section. For each indent, a full load displacement curve with changing time was recoded, and the average changes of strain ε the function of time t are present in Figure 5. Here, the profiles of strain rate $\dot{\varepsilon }$ the function of time are obtained by the following Equation [29]:

(3) $$\dot{\varepsilon }=\frac{\dot{h}}{h}$$

where h is the instantaneous indent displacement and $\dot{h}$ is the displacement rate of the indent tip. The displacement h at a constant load can be obtained by fitting the curve of displacement versus time using an empirical Equation [30]:

(4) $$h=h_{0}+a\cdot t^{b}+c\cdot t$$

where, $h_{0}$ is the indentation depth at the onset of creep, *a*, *b* and *c* are fitting parameters. As shown in Figure 5, after an initial penetration of the indenter into the sample, a short primary creep can be seen followed by a longer and steady state secondary creep stage [29]. Additionally, the strain rate decreases rapidly with time in the primary creep stage and approximately reaches a constant in the second creep stage.

**Figure 6:**
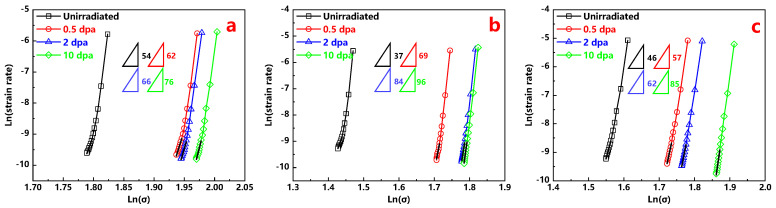
Ln (strain rate) versus ln (Δ) profiles of the (**a**) Hastelloy N alloy; (**b**) Alloy 800H; (**c**) 316H stainless steel are plotted to compute *n* values of secondary stage.

It is widely accepted that the elongation at fracture is controlled by the steady state secondary creep stage, instead of primary creep stage [31]. According to the changes of strain. ε and the strain rate $\dot{\varepsilon }$, the strain at dwell time t < 40 s is not used for further discussion for all the alloys. Hence, the creep in the rest of time belongs to the steady creep stage conforming to the standard creep Equation [32] as follows:

(5) $$\dot{\epsilon }=A\sigma^{n}$$

in the above Equation, n is the stress exponent, *A* is the fitting parameter associate with temperature and material. σ is the applied stress and can be calculated by following Equation [32]:

(6) $$\sigma =\frac{F}{r\times h^{2}}$$

where F is the applied load and parameter *r* is related to the indenter shape. For Berkovich tip, the parameter *r* is 24.5. The profiles of ln (strain rate) versus ln (σ) of all three types of alloys before and after irradiation are shown in Figure 6. The curves are displaced sequentially in order of dose for all the alloys, which means that the applied stress increases at the same indenter depth with the increasing dose. These increments of applied stress reflect irradiation induced hardening as well. Moreover, it is worth noting that the strain rate is decreased with the increasing ion dose at the same applied stress.

**Figure 7:**
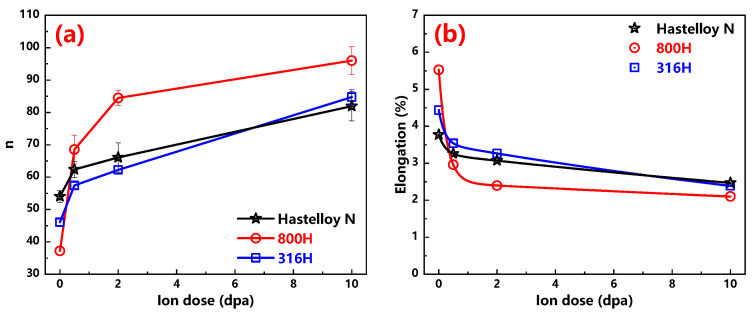
(**a**) *n* values versus ion dose profiles of the Hastelloy N alloy, Alloy 800H, 316H stainless steel are exhibited; (**b**) The elongation versus ion dose of the Hastelloy N alloy, Alloy 800H and 316H stainless steel are presented.

The n value extracted from the slopes of the ln (strain rate) and ln (σ) at dwell time from 40 s to 100 s for all investigated alloys is exhibited in Figure 6 as well. It should be noted that the *n* value of the Alloy 800H at room temperature is estimated to be around 30 ± 15 by linear extrapolation [17]. In this study, the stress exponent n of the unirradiated sample of the Alloy 800H is 37, which is within range of reference reports [17]. As shown in Figure 7a, obviously, the stress exponent values of irradiated samples are bigger than that of unirradiated samples and increases with the increase in ion dose for all the alloys.

It is known that the total elongation obtained in the tensile test is often considered as a measure of plasticity. According to previous studies [33,34], the maximum total elongation is found to correspond closely to the stress exponent *n*. Furthermore, an empirical relation presented by Burkey and Nix for the development of plastic instabilities in tension creep can predict a percentage elongation at failure [35]. This theory ignores the possibility of cavity nucleation. Hence, that, Equation gives an upper limit for the maximum elongation, which is presented in the following Equation:

(7) $$Elongation\left(\%\right)=\frac{k}{n-1}\times 100\%$$

where k is a constant typically equal to 2–3. Here, the value of *k* is 2 for all the alloys. The elongations for all investigated materials before and after irradiation are given in Figure 7b. Previous studies [36,37] have reported that the elongation of the Hastelloy N alloy and Alloy 800H are respectively 42.5% and 58.5% obtained by tensile tests on unirradiated samples at RT. In this study, for these two alloys before irradiation, the calculated elongation values on basis of the nanoindentation results were 3.7% and 5.5%, respectively, this trend is consistent well with the results of tensile tests. It also shows that this method is reasonable to characterize the elongation of materials. If these two sets of elongation values are compared, it is not difficult to find that the latter is one order of magnitude smaller than the former. This means that the ‘true’ elongation values of the materials cannot be obtained using this method. However, it should be emphasized that it is feasible to use these calculated elongation values to compare the relative plasticity of different materials or changes of plasticity to understand the trend. It can be found from Figure 7b that the elongation of the three alloys decreases with the increase of ion dose, and all of them have the tendency of to rapidly decrease first and then slowly decrease. Comparatively speaking, the elongation values of the 316H and Hastelloy N alloy are similar with the variation of ion dose, while those of the Alloy 800H are slightly lower.

For each alloy, the elongation is decreasing with increasing dose, which mainly results from defects introduced by ion irradiation. The reason is discussed as following. Dislocation glide and climb are two main factors of indentation creep [38,39]. Under indent tip, the zone within core will be no creep deformation because there are no shear stresses. However, the zone outside the core in the elastic-plastic zone has deviatoric stress field, and this exert forces on the defects present, such as dislocations, causing creep. Here, the activation energy ΔG required for a dislocation, subjected to overcome the discrete obstacles has been proposed [40]. Under these shear stresses, dislocation gliding meet obstacles which can increase the activation energy ΔG and attempt to bypass or cut through them. If a gliding dislocation is held up by obstacles, dislocation climbing allowing it to glide to the next set of obstacles where the process is repeated [39]. At high temperature, dislocation can climb as well as glide. In this study, the creep tests are performed at room temperature. Thus, the dislocation gliding dominated the indentation creep processes. For the unirradiated samples, the obstacles, such as precipitates, are distributed rarely and evenly. As for the irradiated samples, the obstacles introduced by ion irradiation, especially dislocation loops, perform as the barriers to hinder the dislocations gliding. It is generally accepted that the density of dislocation loops increases along with increment of the irradiation dose, resulting in the increment of the activation energy ΔG for dislocation [40] and the reduction of strain rate, thus increasing stress exponent *n* and degrading creep plasticity.

**Figure 8:**
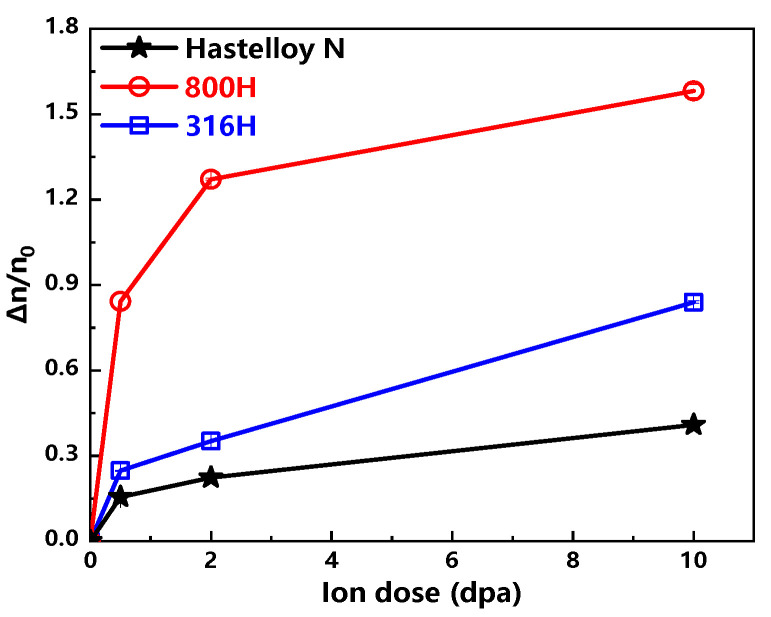
Δnn0 versus ion dose curves of the Hastelloy N alloy, Alloy 800H and 316H stainless steel are displayed.

In view that the elongations of all the unirradiated samples are different, the ratios of stress exponent increasement Δn and $n_{0}$ (the stress exponent of unirradiated sample) are calculated to assess the changes of creep properties for all investigated alloys as shown in Figure 8. Similar with the changes of irradiation induced hardening, the smallest change of stress exponent *n* is the Hastelloy N alloy, followed by the 316H stainless steel and Alloy 800H, which means that the Hastelloy N alloy is the most irradiation resistance among these three alloys.

**Figure 9:**
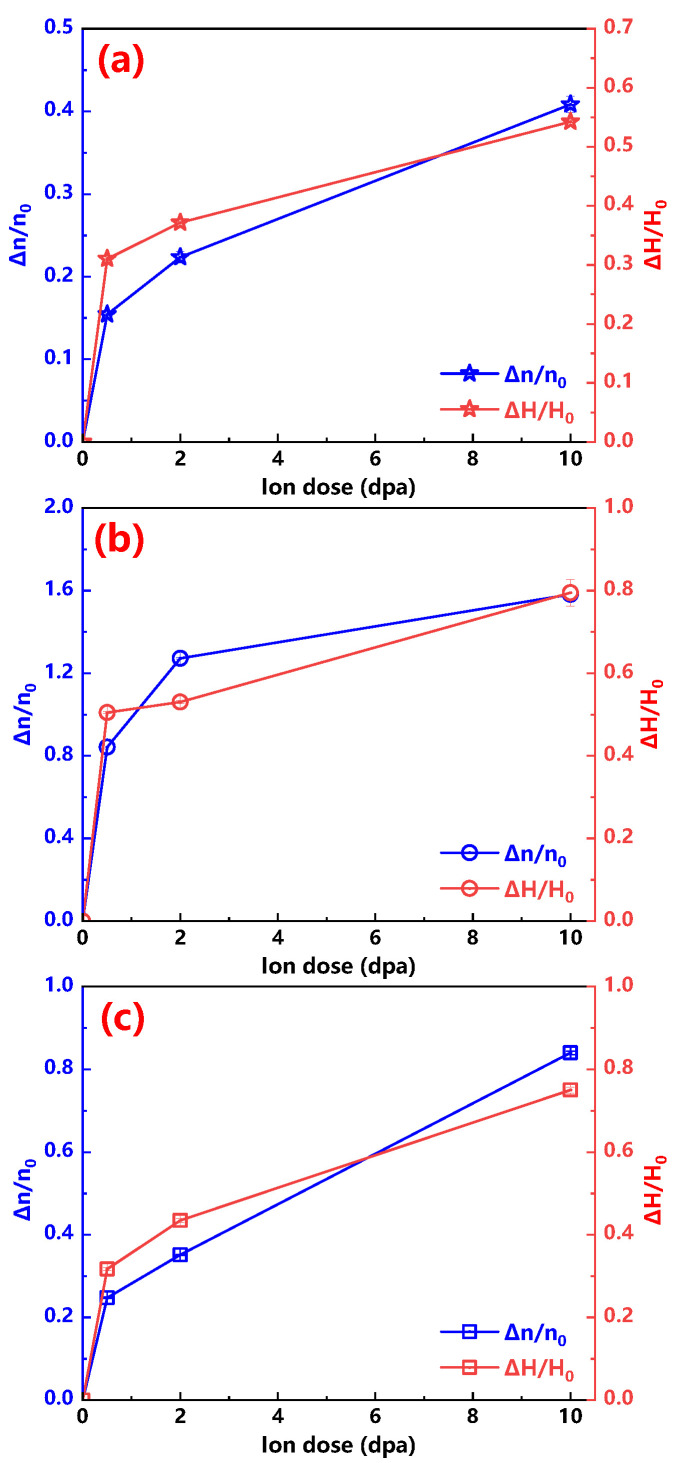
ΔHH0 and Δnn0 versus ion dose profiles of the (**a**) Hastelloy N alloy; (**b**) Alloy 800H; (**c**) 316H stainless steel are shown.

Currently, it is widely accepted that characterizing the irradiation induced hardening can illustrate the irradiation embrittlement, but few studies confirm whether the irradiation induced hardening consists with the irradiation embrittlement or creep plasticity degradation. In this study, both creep plasticity degradation and irradiation induced hardening of all investigated alloys are characterized, and their degrees of change are present in Figure 9. It is noteworthy that the variation trends of irradiation induced hardening and creep plasticity degradation are almost identical, which indicates that the change of hardness for heavy ion irradiated alloy may be used to evaluate the change trend of creep plasticity.

## 4. Conclusions

In this study, the Hastelloy N alloy, Alloy 800H and 316H stainless steel were irradiated by Xe^20+^ ion with energy of 4 MeV to 10 dpa at RT. Their micromechanical properties, hardness and creep plasticity, are characterized by nanoindentation at room temperature to reveal the irradiation resistance of the Alloy 800H and 316H stainless steel by comparison with the Hastelloy N alloy. Additionally, the relationship between irradiation induced hardening and creep plasticity degradation is discussed. The main conclusions are as follows:

1. The hardening phenomenon occurs in the irradiated samples and hardness increases with increasing ion dose up to 10 dpa in all the alloys. Among them, the best irradiation hardening resistance appeared in the Hastelloy N alloy, followed by 316H stainless steel and Alloy 800H;
2. The stress exponent *n* increases with the increasing ion dose in all the alloys, which shows that plasticity of all three alloys degraded after irradiation. By comparison, the Hastelloy N alloy is evaluated to process good irradiation resistance, whereas that of the Alloy 800H and 316H is slightly worse;
3. The variation trends of irradiation induced hardening and stress exponent increase are almost identical. The results show that the property of creep plasticity of irradiated materials can be reflected from the nanohardness measurement for the heavy ion irradiation cases.

## Figures and Tables

**Table 1:** Nominal material compositions (wt.%) of the Hastelloy N alloy, Alloy 800H and 316H stainless steel.

Elements	Ni	Mo	Cr	Fe	Mn	Si	C	Ti	Al	Co
Hastelloy N	Bal.	16.5	6.96	4.2	0.71	0.46	0.05	≤0.2		≤0.02
800H	30.4	–	20.1	47.8	0.8	0.3	0.08	0.26	0.26	–
316H	12.5	1.4	18.4	Bal.	2.1	1.2	0.3	–	–	1.4
